# Round the Hospitals

**Published:** 1917-03-31

**Authors:** 


### ROUND THE HOSPITALS.

Among the treasures to be offered at the Red Cross Sale at Messrs. Christie's there will be disposed of, on Wednesday, April 4, some letters of Florence Nightingale's, and a small leather writing-case with an inscription by her, and also a little note enclosed, dated September 1, 1896, which runs: "God bless our departing Home Sister, Miss Crossland, says every petal of these poor flowers and every grape of the vine." It is evident that flowers and fruit had accompanied the gift of the writing-case. Of the letters perhaps the most interesting is one dated "Castle Hospital, Balaclava, Nov. 21st, 1855," and addressed to the Officer of Transports. In this Miss Nightingale states that an outbreak of cholera has occurred at Scutari, and asks for papers to enable her to go there, "her return there being urgently desired." It would be interesting to know if the noble request was granted.

Two interesting events have marked the present week at St. Mary's Hospital, Paddington. One was the annual meeting of the Ladies' Work Association, when more than 300 articles were presented for the use of the patients. Instead of the usual show of work the wards were visited and an inspection made of the work done by the wounded soldiers, which created deep interest and some admiration. The patron saint of St. Mary's Hospital was commemorated by a special service on the 26th instant, with Holy Communion at 6 a.m., Evensong and a sermon preached by Archdeacon Holmes at 5.30 p.m. The service was attended by the nursing staff and patients, H.R.H. the Princess Arthur of Connaught having a seat in the choir stalls, where she was attended by the Home Sister, Miss Morrison.

The Archdeacon's text was "The Soldiers," who he remarked were now everywhere. Three things, the Sword, the Sash, and the Salute, linked our thoughts with the soldier. A visit to the Tower reveals the sword of justice with its sharp edge, and the sword of mercy with its blunt edge, whilst the crossed swords are the emblem of St. Paul's Cathedral. The sash in olden times was used for carrying wounded men from the battlefield, a fact which marks the enormous developments in ambulance and transport methods, whereby a wounded man is now brought from the battlefield in France to a London hospital in a relatively few hours. The ambulance service from the railway stations in the Homeland to the hospitals is notably efficient, as are the services and work of the stretcher-bearers in this connection, and also at the Front. The salute now witnessed in the streets everywhere is of two kinds, the officers' and the privates'. Both are symbolical. There is a sign of a real belief in a real Jesus in a salute. The private raises the hand on a level with the eyebrows; this comes from the old tournament custom where the knight used to raise his hand to shade his eyes from the beauty of those of his lady. Chivalry men owe and must show to women, whilst women for their part should demand this mark of respect. The Archdeacon made a good point by insisting that everybody's respect and recognition are due to our Homeland Nurses, even more than to the nurses who rush off to the Front. This is so because the Homeland Nurse has refused the temptation to seek excitement abroad and has remained loyal to her duty, done without honour or glory, from a high sense of responsibility and loyal service to the sick.

That the domestic staffs of hospitals and institutions are affected by the present unrest in the ranks of domestic workers generally, which leads them to migrate to fresh fields of work, has become evident for some time. The Brentford Council have offered bonuses of £5 to the cook, wardmaid, and housemaid at the hospital, if they will remain until after the war. Approval has been given by the Army Council for a war bonus of £5 5s. a year, with effect from January 1, 1917, to be paid to the cook, the matron's maid, and the senior mess-room maid of the Royal Hospital, Chelsea. The authorities of St. Bartholomew's Hospital have laid down a new scale of pay for their domestic staff. The commencing wages for wardmaids, dining-room maids, housemaids, and kitchenmaids are to be £16 instead of £15, increasing by £1 per annum to a maximum of £22, with a bonus of £3 on completion of the first three years' service. The senior maids, according to their class, are to begin at wages varying from (*a*) £20 to £25, (*b*) £18 to £22, (*c*) £22 to £25, and (*d*) £20 to £23, by yearly increases of £1, with a bonus of £3 on completion of the first three years' service. Further, all maids who have been in the service of the hospital over three years are to have an immediate increase of wages of £1, or if over five years of £2, with subsequent increases to date accordingly. In place of the £2 war bonus agreed to be paid on the termination of the war to servants of over one year's service, a special war bonus of £1 is to be given on the completion of each six months' service commencing from January 1, 1917. It is calculated that the annual increased cost to St. Bartholomew's Hospital of this new scale, including the payment of war bonuses, will be £245 on the present staff of eighty-three maids.

The nursing staff of the King George Hospital in Stamford Street, S.E., are providing a memorial to those connected with the hospital or who are residents in the parish of St. John, Waterloo Road, who have fallen in the war. The ambitious character of this memorial will be gathered from the fact that, the monument takes the form of a lofty pillar of stone, surmounted by a life-size bronze figure of Christ, which will be placed within the precincts of St. John's Church, facing Waterloo Road. May we conclude that pride, as workers in The King George Hospital of the nation, has fired the ambition of the nursing staff to take this lofty

For "Food in Wartime," see p. 524; "Patriotism in Nurse-Training Schools," see p. 525;
Matrons' and Sisters' Appointments, see p. iv.

**ROUND THE HOSPITALS**—(*continued*).

flight. Hence this decision to make their memorial remarkable among all those which are being erected up and down the country?

We are glad to note that the *Daily Mail* has allowed an Army Reserve Sister to explode the criticisms on hospital nurses' food which have appeared in that paper, by insisting that they must refer to small hospitals, seeing that the nurses' food at the military hospitals is both varied and excellent in quality, as well as sufficient in quantity. This sister adds, she often wonders how the nurses in military hospitals can get such a diet, seeing that as a probationer in a civil hospital fifteen years ago, when food was cheap, she did not get either the quantity or quality of food at present supplied in the Army.

No time has been lost in following the advice of Mrs. Tennant, given at the National Service meeting, to collect scraps of woollen and cotton fabrics for re-manufacture. Already in some of the suburbs of London "Rally Leagues" have been formed for the house-to-house collection of suitable rags. We do not know if hospitals are to be included in the collection, but there, where "condemning day" is a regular feature, there must be many oddments which might be given to the League. As the price received from the manufacturers for the rags is to be given to the Red Cross funds, this contribution of "shreds and patches" forms a means by which even the poorest and humblest may contribute to the war. But one word of warning may be given. With measles rampant at present it behoves every donor to see that whatever she gives is thoroughly clean. Many people may think that as the rags are not to be worn, but torn up and made over, absolute cleanliness is not of the first importance. But we are sure that if they give the matter a thought every woman will see to it that her contribution, be it large or small, has no chance of infecting those who receive or have to work with the rags. In former days rag-sorters' disease was not uncommon. It would be indeed grievous if anything of the kind was associated with gifts meant for national service.

